# Report on Sanitary and Quarantine Regulations

**Published:** 1890-11

**Authors:** 


REPORT AND RECOMMENDATIONS CONCERNING SANITARY AND QUARANTINE REGULATIONS IN COMMERCE WITH THE AMERICAN REPUBLICS.

This report is upon a means of so harmonizing measures for the prevention of the spread of disease that there shall be no conflict between the diverse sanitary regulations which the American nations have adopted to protect themselves from these infectious diseases.

We quote a significant paragraph:

"Complete isolation, which theoretically appears to be the most effectual prophylactic against the invasions of epidemic diseases, does not afford in practice satisfactory results as a sanitary measure, but tends, on the other hand, to notably injure the commercial interests of the countries. The distinguished professor, Dr. Francisco Rosas, President of the Sanitary Congress of Lima, thus expresses himself on this point: 'It is scientifically demonstrated by innumerable facts that the closing of ports and frontiers does not prevent the invasion of epidemics, that these enter and develop with greater violence in the countries which pretend to isolate themselves, because, under the mistaken belief that they are free of all danger, they disregard the proper means to restrain the development of the epidemic and, above all, to lessen its severity.' "

The reader should now refer to Dr. T. Dwight Stow's paper on contagion in THE HOMŒOPATHIC PHYSICIAN for February, 1888, page 49. The pamphlet then gives the text of a treaty for the regulation of the quarantine practices between the several countries, reducing them to a uniform code.

